# Four Prognosis-Associated lncRNAs Serve as Biomarkers in Ovarian Cancer

**DOI:** 10.3389/fgene.2021.672674

**Published:** 2021-07-02

**Authors:** Jianfeng Zheng, Jialu Guo, Huizhi Zhang, Benben Cao, Guomin Xu, Zhifen Zhang, Jinyi Tong

**Affiliations:** ^1^Department of Obstetrics and Gynecology, Affiliated Hangzhou Hospital, Nanjing Medical University, Hangzhou, China; ^2^Department of Obstetrics and Gynecology, Hangzhou Women's Hospital, Hangzhou, China; ^3^Department of Fourth Clinical Medical College, Zhejiang Chinese Medical University, Hangzhou, China; ^4^Department of Obstetrics and Gynecology, Haining Second People's Hospital, Haining, China

**Keywords:** ovarian cancer, lncRNA, weighted gene coexpression analysis, risk score model, cell function assays

## Abstract

Long non-coding RNAs (lncRNAs) play crucial roles in ovarian cancer (OC) development. However, prognosis-associated lncRNAs (PALs) for OC have not been completely elucidated. Our study aimed to identify the PAL signature of OC. A total of 663 differentially expressed lncRNAs were identified in the databases. According to the weighted gene coexpression analysis, the highly correlated genes were clustered into seven modules related to the clinical phenotype of OC. A total of 25 lncRNAs that were significantly related to overall survival were screened based on univariate Cox regression analysis. The prognostic risk model constructed contained seven PALs based on the parameter λ_min_, which could stratify OC patients into two risk groups. The results showed that the risk groups had different overall survival rates in both The Cancer Genome Atlas (TCGA) and two verified Gene Expression Omnibus (GEO) databases. Univariate and multivariate Cox regression analyses confirmed that the risk model was an independent risk factor for OC. Gene enrichment analysis revealed that the identified genes were involved in some pathways of malignancy. The competitive endogenous RNA (ceRNA) network included five PALs, of which four were selected for cell function assays. The four PALs were downregulated in 33 collected OC tissues and 3 OC cell lines relative to the control. They were shown to regulate the proliferative, migratory, and invasive potential of OC cells via Cell Counting Kit-8 (CCK-8) and transwell assays. Our study fills the gaps of the four PALs in OC, which are worthy of further study.

## Introduction

Ovarian cancer (OC) is a gynecological malignancy with the highest morbidity and mortality rates worldwide (Stewart et al., 2019). Although the prognosis of patients in early cancer stages is better, most patients are already in the late stages of OC during the first diagnosis (Kaldawy et al., 2016; Eisenhauer, 2017). Thus, there is a need to identify novel biomarkers for predicting tumorigenesis and clinical diagnosis in earlier stages and to develop new therapeutic strategies and targets for OC.

Long non-coding RNAs (lncRNAs) are endogenous RNA transcripts more than 200 nucleotides in length that are not translated into polypeptides (Kopp and Mendell, 2018). Previous studies have found that lncRNAs could serve as strong prognostic biomarkers and play an important role in

different cell processes such as cell migration, growth, invasion, apoptosis, and differentiation in OC (Wang et al., 2019). For example, *SNHG9* was shown to serve as an anticancer biomarker by regulating *miR-214-5p* (Chen et al., 2021). *LINC01969* was confirmed to be a cancer-promoting biological marker via the *miR-144-5p*/*LARP1* axis (Chen et al., 2020).

The pathogenesis of most cancers, including OC, are caused by various genes rather than a single gene (Van Cott, 2020). The risk model can estimate a set of risk genes in any given cancer (Cintolo-Gonzalez et al., 2017; Tammemägi et al., 2019). Weighted correlation network analysis (WGCNA) is a combined method for analyzing clinical information and gene expression data (Sun et al., 2017). Through the analysis of gene modules with high correlation with clinical information, a series of key genes with high connectivity in the modules were obtained, which are potentially important for the occurrence and development of tumors (Tian et al., 2017). Previous studies have identified lncRNA-based signatures via WGCNA or by developing a risk model for OC (Li and Zhan, 2019; Zhao and Fan, 2019). However, these studies are still limited in their lack of cell function assays and constantly updated databases.

Thereby, the purpose of our research was to identify a prognosis-associated lncRNA (PAL) signature serving as a noteworthy prognostic biomarker in OC by using WGCNA and other comprehensive analyses. Two additional datasets from the GEO were used to check the accuracy of the model. A competitive endogenous RNA (ceRNA) network was established to explore the mechanisms of candidate PALs. Finally, four candidate PALs were selected for the *in vitro* assays.

## Materials and Methods

### Data Extraction and Pre-treatment

The RNA data with corresponding clinical information were downloaded from TCGA TARGET GTEx (https://toil.xenahubs.net) (Goldman et al., 2020) and GEO (GSE32063 and GSE17260, https://www.ncbi.nlm.nih.gov/geo/) (Yoshihara et al., 2010, 2012). RNAs with expression levels >0 in 33% of the samples were identified as messenger RNAs (mRNAs) or lncRNAs based on annotation information from the GENCODE database (https://www.gencodegenes.org/) (Harrow et al., 2012). Differential gene analysis was based on linear regression and empirical Bayes using the limma package (http://www.bioconductor.org/packages/2.9/bioc/html/limma.html) (Ritchie et al., 2015). Meanwhile, we evaluated the differences in multiple and significance levels using Benjamini and Hochberg multiple comparisons (*P* < 0.05, |logFC| > 2).

### Screening Modules Related to Clinical Phenotype

WGCNA considers not only the coexpression patterns between two genes but also the overlap of neighboring genes (Langfelder and Horvath, 2008). A coexpression network between differentially expressed mRNAs and lncRNAs was established using WGCNA from the R package to identify modular genes closely related to the clinical phenotype. Clinical phenotypes in our study included age at initial pathological diagnosis, clinical stage (stage I, II, III, or IV), lymphatic invasion (no or yes), neoplasm histologic grade (grade I, II, III, or IV), tumor residual disease (no macroscopic disease, 1–10 mm, 11–20 mm, or >20 mm), venous invasion (no or yes), and vital status (dead or alive).

The WCGNA analysis should be subject to scale-free networks. Therefore, the applicable weight parameter β (SoftPower) of the gene coexpression matrix was supposed to conform to the scale-free distribution to the maximum extent. The correlation coefficients (R) of connectivity k and p(k) under each β were calculated, and then, β was selected when *R*^2^ reached 0.85 for the first time. The highly correlated genes were clustered into modules based on clustering and dynamic pruning methods (minModuleSize = 30; MEDissThres = 0.3). Finally, the gene assembly modules closely related to the phenotype were identified via the correlation between the module and clinical phenotype.

### Construction and Validation of Risk Model

The lncRNAs in the aforementioned modules were analyzed by univariate Cox regression analysis based on their expression values and overall survival (OS) of each OC sample (*P* < 0.05). Kaplan–Meier (K–M) analysis and log-rank test were performed using the R package to select the PALs (*P* < 0.05) for further analysis. Least absolute shrinkage and selection operator (Lasso) regression of the glmnet package (version 2.0-18) (Engebretsen and Bohlin, 2019) was carried out for further dimensionality reduction to screen the more significant PALs for risk model construction. According to multivariate Cox regression analysis, a prognostic risk model was generated based on the following formula:

$$\begin{array}{l} Risk\;score=\sum \beta_{\text{lncRNA}}\;\times \;Exp_{\text{lncRNA}} \end{array}$$

In the risk score (RS) formula, β_lncRNA_ represents the regression coefficient for PALs, and Exp_lncRNA_ means the expression level of homologous PALs. OC patients in the study were divided into low- or high-risk groups according to the optimal cutoff point of RSs gained from Survminer (version 0.4.3) from the R package, and K–M survival analysis was performed between the two risk groups using the log-rank test. In addition, GSE32063 (40 OC samples) and GSE17260 (110 OC samples) were downloaded from National Center for Biotechnology Information (NCBI) GEO (Barrett et al., 2005) and used to develop a prognostic risk model using the same method.

Furthermore, the RSs of different clinical indicators, including age (age ≤ 60 years or >60 years), grade (grade II or III), and stage (stage III or IV) were compared. Several clinical indicators, such as age in GEO, grade I, grade IV, stage I, and stage II, were excluded due to insufficient sample size. Tumor mutational burden (TMB) scores were calculated for each OC patient from TCGA, and the relationship between the risk model and TMB was also assessed.

### Construction of ceRNA Network

The microRNAs (miRNAs) targeted by the corresponding PALs were speculated by the DIANA-LncBase v2 (Paraskevopoulou et al., 2016). The target mRNAs by the corresponding miRNAs were speculated using miRTarBase (Hsu et al., 2011). Subsequently, the ceRNA network based on the same miRNAs of PAL–miRNA and miRNA–mRNA was constructed and visualized using Cytoscape (Kohl et al., 2011).

### Biofunctional Analysis

Kyoto Encyclopedia of Genes and Genomes (KEGG) analysis was performed on potential target mRNAs of clinical modules or PALs based on clusterProfiler of R package (version:3.8.1, pAdjustMethod = BH, pvalueCutoff = 0.05) (Yu et al., 2012).

### *In vitro* Assays

With approval from the Ethics Committee, a total of 33 OC and 20 adjacent normal ovarian tissues were collected from surgery OC patients between May 2019 and January 2021 at the Affiliated Hangzhou Hospital of Nanjing Medical University (Hangzhou, China). The clinical features are shown in Supplementary Table 1. The OC cell line SKOV-3 was supplied by the Cell Bank of China Academic of Science (Shanghai, China). The IOSE-80, HO-8910, and A2780 cells were purchased from iCell Bioscience Inc. (Shanghai, China). SKOV-3 cells were cultured in McCoy's 5A medium supplemented with 10% fetal bovine serum (FBS) and 1% penicillin-streptomycin. IOSE-80, HO-8910, and A2780 cells were cultured in 90% Roswell Park Memorial Institute (RPMI) 1640 medium with 10% FBS and 1% penicillin-streptomycin. All cells were incubated in a 5% CO_2_ incubator at 37°C.

After RNA extraction and reverse transcription, real-time quantitative PCR (qPCR) analysis was performed using an ABI 7500 instrument to evaluate the expression value of alternative PALs in cells and tissues based on the kit of Takara (Shiga, Japan). Primer sequences are listed in Table 1.

**Table 1:** The primer sequences in PCR analysis.

**Symbol**	**Sequences (5^**′**^-3^**′**^)**
hGAPDH-F	GTCAACGGATTTGGTCTGTATT
hGAPDH-R	AGTCTTCTGGGTGGCAGTGAT
hTCL6-F	ACCATCCCAAAGCCAACG
hTCL6-R	AAGTCATAAGGAACGGCATAAA
hVLDLR-AS1-F	TCATCACAGCATCCTTCACAGCC
hVLDLR-AS1-R	AACAAGCCACACTGACAGACCAT
hRP11-356I2.4-F	AGCCTTGTTGCCACGGAGAC
hRP11-356I2.4-R	ACGCATGACGCACAGAAGAGT
hLINC00893-F	GCTGCTCCTCACTCTCACTCCT
hLINC00893-R	CCTCTCCTCATCCGACCACAGA

The plasmids used for the experiment were constructed by TSINGKE Biological Technology (Hangzhou, China) based on the known sequences of *TCL6, VLDLR-AS1, RP11-356I2.4*, and *LINC00893* from NCBI. SKOV-3 and HO-8910 cells were transfected with homologous plasmids (pcDNA-NC, pcDNA-TCL6, pcDNA-VLDLR-AS1, pcDNA-RP11-356I2.4, and pcDNA-LINC00893) using the jetPRIME transfection reagent (Polyplus Transfection, Shanghai, China), according to the manufacturer's instructions.

Subsequently, transfected SKOV-3 and HO-8910 cells were made into cell suspensions and then transferred to a 96-well plate or upper chamber (with or without Matrigel) of 24-well transwell inserts (8 μm pore size). For the Cell Counting Kit-8 (CCK-8) assays, the old culture media were removed, and 10 μl cell counting kit-8 solution (MedChemExpress, China) with 90 μl media was added to each well for an additional 2 h on days 1–4. At the wavelength of 450 nm, the OD value of each well was detected by a spectrophotometer (Thermo Scientific, Massachusetts, America). For the transwell assays, the lower chambers were added with 500 μl medium containing 30% FBS. The bottoms of the upper chamber were fixed with 4% paraformaldehyde and stained with crystal violet for 10 min on day 1. The number of cells that invaded through the membrane to the lower surface was counted using Image J software after photographing using a microscope.

The experiments were conducted in triplicate, and each experiment was repeated three times. Statistical analysis was performed using GraphPad Prism version 8.0.1. The data were analyzed by Student's *t*-test or one-way analysis of variance (ANOVA). *P* < 0.05 was considered statistically significant.

## Results

**Figure 1:**
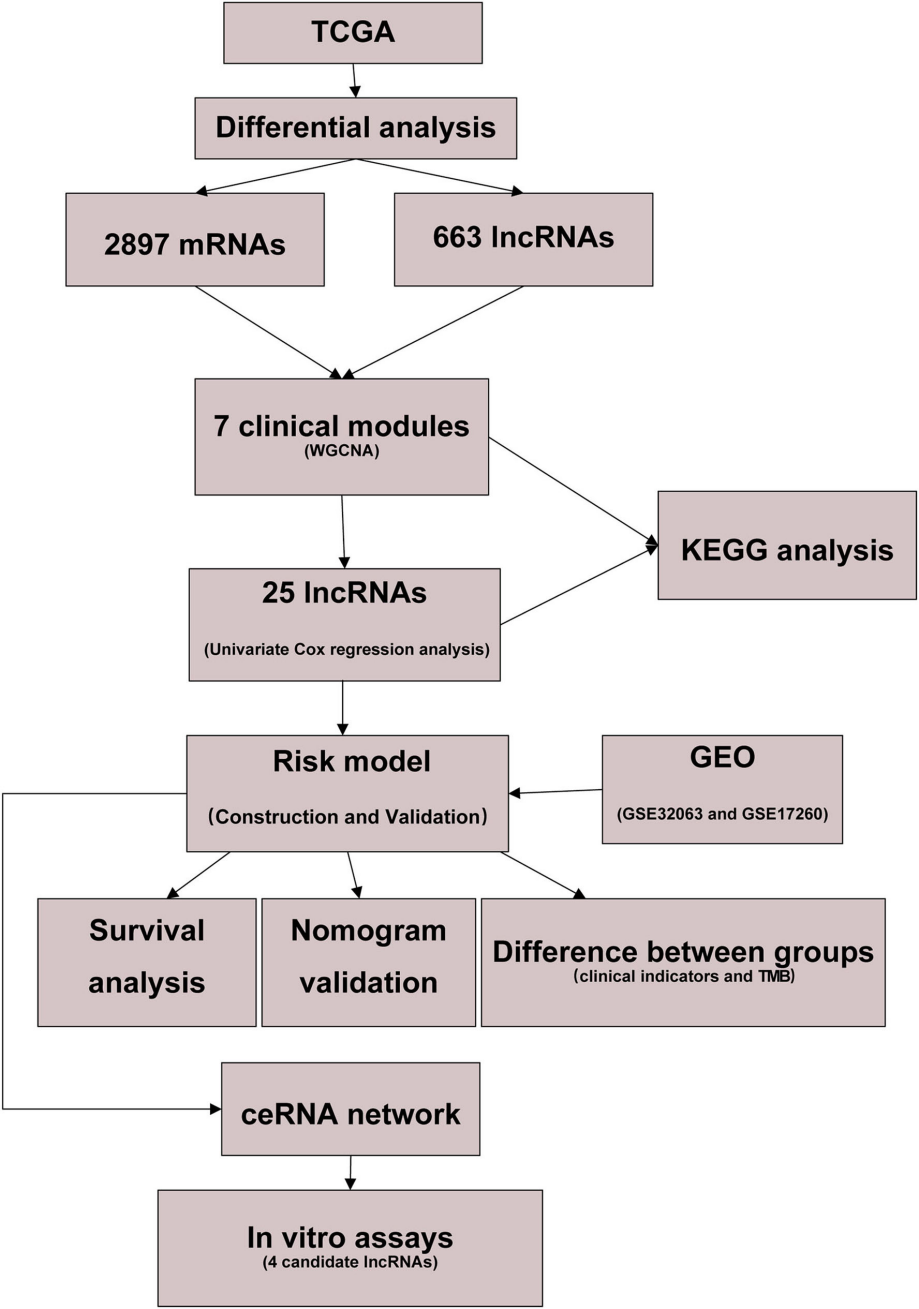
Flow diagram of our study.

To facilitate the understanding of our entire study, we created a flowchart, which is shown in Figure 1.

### Screening Modules Related to Clinical Phenotype

**Figure 2:**
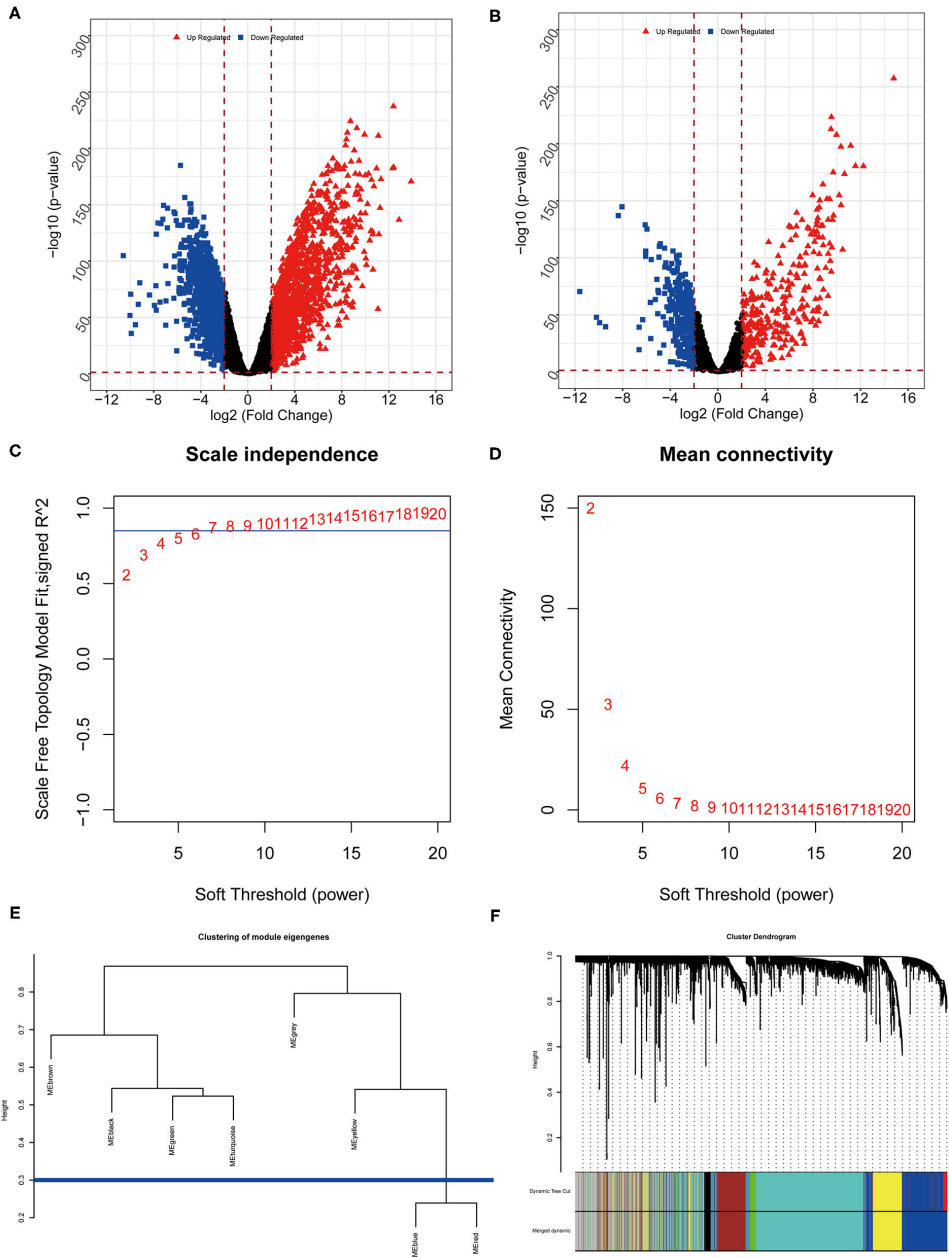
Results of differential analysis and WGCNA. **(A)** Volcano map of differential mRNAs. **(B)** Volcano map of differential lncRNAs. **(C)** Selection graphs of β. **(D)** Schematic diagram of the mean connectivity. **(E)** The module clustering result diagram. The vertical axis represents the difference coefficient, and the blue line represents the difference coefficient of 0.3. **(F)** Systematic cluster tree of genes and gene modules generated by dynamic clipping method.

After the difference analysis, a total of 1,467 upregulated mRNAs and 1,431 downregulated mRNAs were extracted (Figure 2A), and 307 lncRNAs expressed at high levels and 356 lncRNAs expressed at low levels were obtained (Figure 2B).

WGCNA analysis was further conducted based on 3,560 differentially expressed genes to screen modules related to clinical phenotypes. We assigned the β value to 7 when *R*^2^ was first ~0.85, which ensured that the network connection was close to the scale-free distribution and was the minimum threshold for smoothing the curve (Figures 2C,D). The modules with correlation coefficients >0.7 (the divergence coefficient was <0.3) were consolidated after clustering (Figure 2E). A total of seven modules (M1-yellow, M2-black, M3-green, M4-brown, M5-blue, M6-turquoise, and M7-gray) were integrated, and the gray module could not be gathered into other modules; hence, the gray module would not be considered in subsequent analysis (Figure 2F).

Two further methods were used to mine the modules associated with clinical phenotypes. First, the correlation between each module feature vector gene and the clinical phenotype was calculated. The feature vector gene was the first principal component gene E of a specific module, which represents the overall level of gene expression in the module. Second, the absolute value of the correlation between gene expression in each module and the clinical phenotype was taken as the correlation between the module and the clinical phenotype (Supplementary Figure 1).

**Figure 3:**
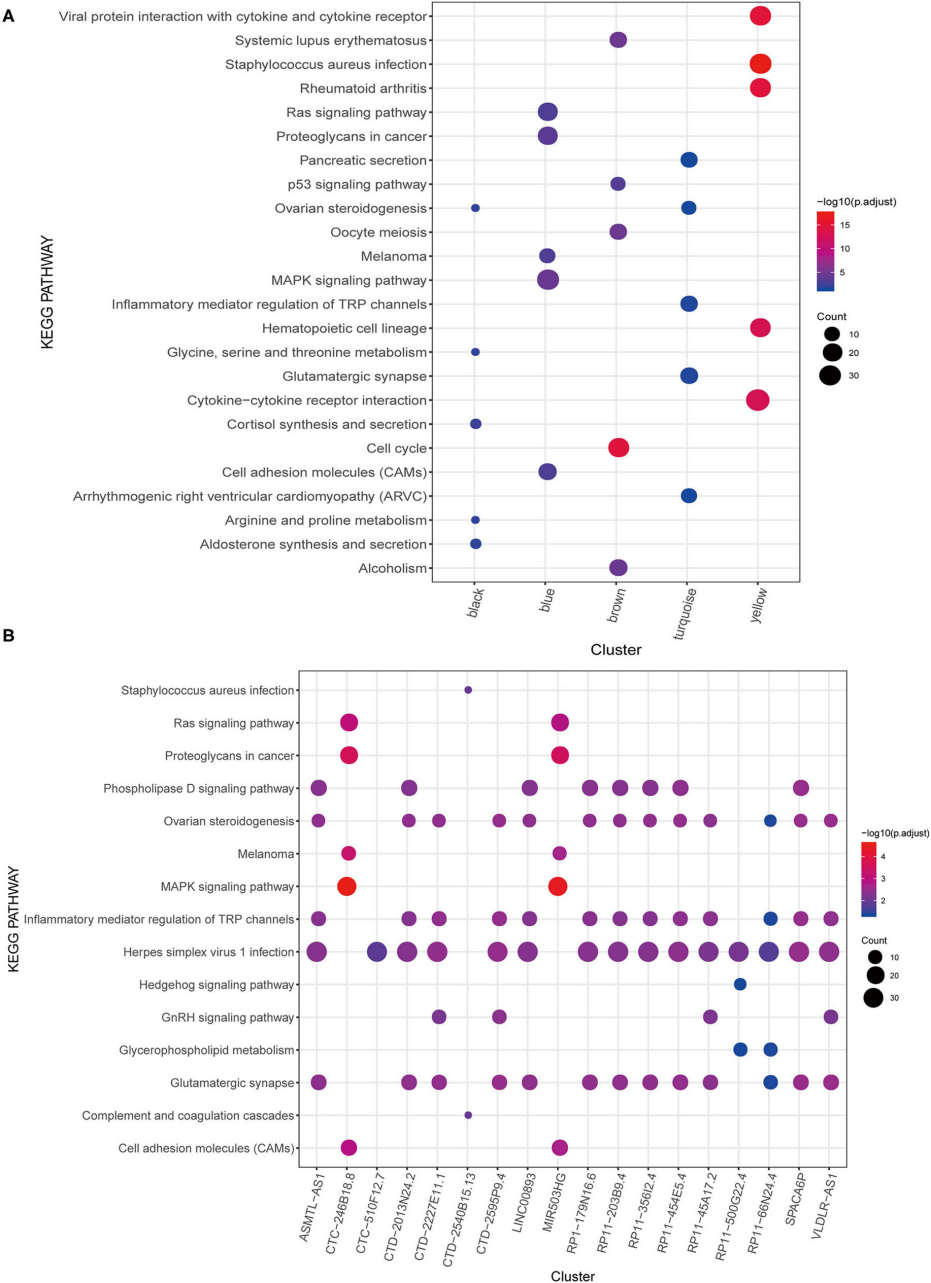
KEGG pathway enrichment analysis. **(A)** WGCNA modules. **(B)** lncRNAs.

Furthermore, the mRNA in each module was subjected to KEGG pathway enrichment analysis, which showed that the black, blue, brown, turquoise, and yellow modules were significantly enriched in 13, 21, 11, 5, and 48 KEGG pathways. Nonetheless, no significant pathways were enriched in the green module. The KEGG pathway of each module was ranked according to the *P*-value, and the top five were selected for display (Figure 3A). Some of these modules are associated with important biological processes in tumor genesis and development, such as Ras signaling pathway and mitogen-activated protein kinase (MAPK) signaling pathway of the blue module, p53 signaling pathway of the brown module, and inflammatory mediator regulation of TRP channels in the turquoise module.

### Construction and Validation of Risk Model

A total of 25 PALs that were significantly associated with OS were screened based on univariate Cox regression analysis, including one upregulated lncRNA (HR > 1) and 24 downregulated lncRNAs (HR <1) (Table 2; Supplementary Figure 2). Among the 25 PALs, KEGG pathways were enriched to 19 lncRNAs, and the top five pathways were selected for display (Figure 3B). Some PALs were enriched in several classic signaling pathways of tumors, such as Ras signaling pathway, MAPK signaling pathway, Hedgehog signaling pathway, and so on. Interestingly, several lncRNAs were enriched in ovarian steroidogenesis or the GnRH signaling pathway (VLDLR-AS1, RP11-356I2.4, LINC00893, and so on) in OC.

**Table 2:** Kaplan–Meier survival analysis of 25 lncRNAs.

**Gene**	**HR**	**Lower 0.95**	**Upper 0.95**	***P-value***	**Type**	**Moudle**
CH507-254M2.2	0.89782612	0.84229443	0.95701896	0.00093768	down_lnc	brown
RP1-179N16.6	0.86639416	0.79455643	0.9447269	0.00116431	down_lnc	turquoise
CTD-2595P9.4	0.93999785	0.90293072	0.97858666	0.00257416	down_lnc	turquoise
RP11-386G11.10	0.92534363	0.87642424	0.97699356	0.00511256	down_lnc	blue
RP11-500G22.4	0.95173106	0.91827361	0.98640754	0.00673863	down_lnc	turquoise
RP11-356I2.4	0.86427736	0.77671338	0.96171302	0.0074451	down_lnc	turquoise
CTD-2227E11.1	0.9271007	0.87607788	0.98109509	0.00877255	down_lnc	turquoise
TCL6	0.94990532	0.9140781	0.98713678	0.0087936	down_lnc	brown
CTC-564N23.2	0.923453	0.8685364	0.98184193	0.01090358	down_lnc	yellow
LINC00893	0.89868222	0.82738124	0.97612767	0.01131416	down_lnc	turquoise
RP5-1180E21.5	0.96699305	0.94185858	0.99279825	0.0124944	down_lnc	turquoise
RP11-66N24.4	0.88211727	0.79714411	0.97614831	0.01522047	down_lnc	turquoise
RP11-454E5.4	0.87249941	0.78102511	0.97468725	0.01579247	down_lnc	turquoise
CTC-246B18.8	1.08022122	1.01397626	1.15079409	0.01685716	up_lnc	blue
MIR503HG	0.91727562	0.85356375	0.98574308	0.01872699	down_lnc	blue
CTD-2013N24.2	0.91086867	0.84264021	0.98462159	0.01876848	down_lnc	turquoise
RP13-39P12.3	0.91678305	0.85080369	0.98787908	0.02260895	down_lnc	turquoise
CTD-2540B15.13	0.89876543	0.81847421	0.9869331	0.02538823	down_lnc	yellow
CTC-510F12.7	0.90586195	0.83058035	0.98796687	0.02551983	down_lnc	turquoise
RP11-254F7.3	0.94196846	0.89291289	0.99371909	0.02846161	down_lnc	turquoise
VLDLR-AS1	0.92027511	0.85239314	0.99356299	0.03357438	down_lnc	turquoise
SPACA6P	0.88785293	0.79493949	0.9916262	0.03493912	down_lnc	turquoise
RP11-45A17.2	0.94046304	0.8881824	0.99582105	0.03542486	down_lnc	turquoise
RP11-203B9.4	0.88611018	0.79063991	0.99310854	0.03763039	down_lnc	turquoise
ASMTL-AS1	0.92770067	0.86065926	0.99996429	0.04989104	down_lnc	turquoise

**Figure 4:**
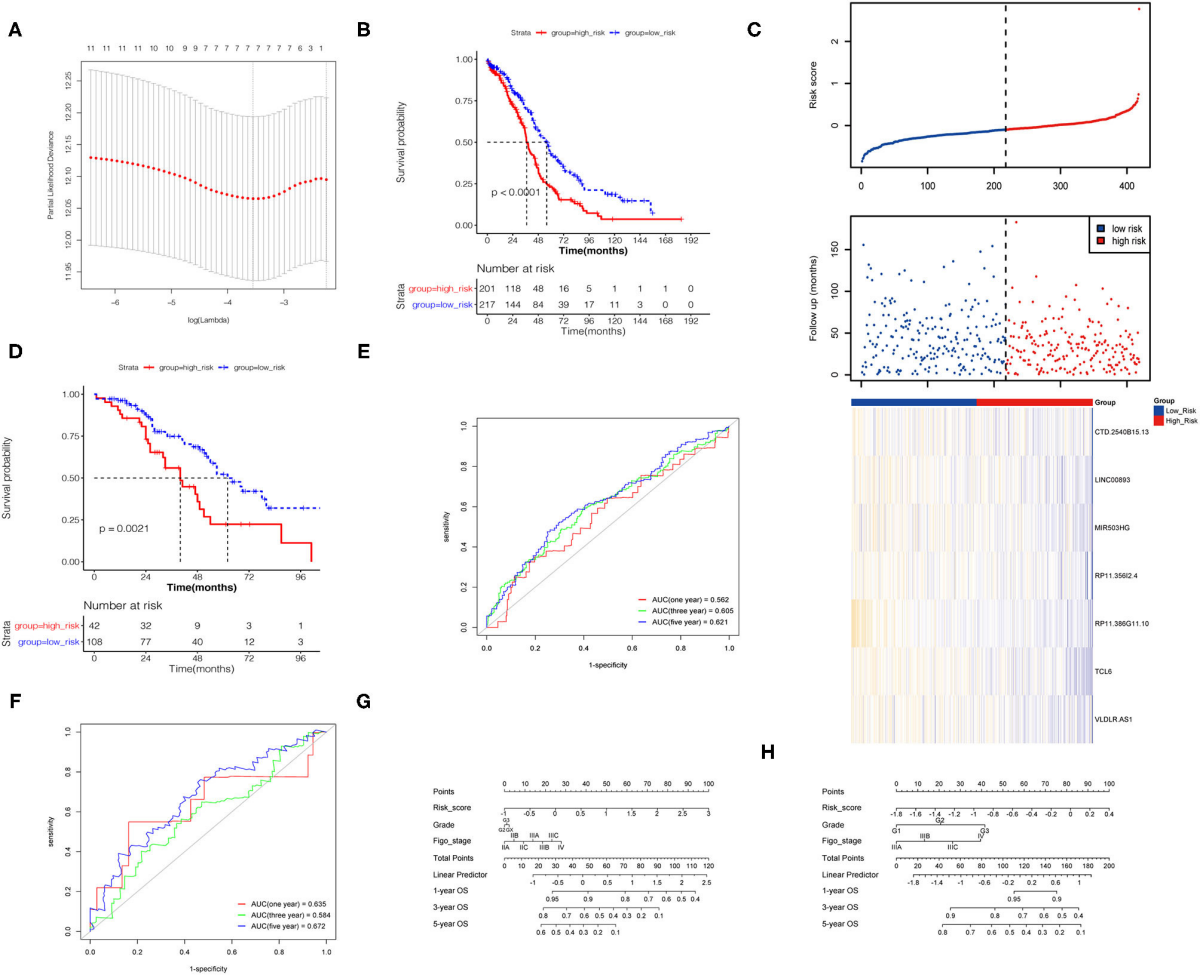
Construction of the prognostic risk model. **(A)** The λ selection diagram in the Lasso model. **(B)** K–M survival curves of high- and low-risk groups for TCGA database. **(C)** Risk score distribution and lncRNA expression heat map for TCGA database. **(D)** K–M survival curves of high- and low-risk groups for GEO database. **(E,F)** Time-dependent receiver operating characteristic (ROC) curve for predicting OS of the risk model. **(G,H)** The nomogram based on the signature and clinical information.

Lasso regression was performed on the above 25 PALs to determine the optimal modeling parameter (λ) (Figure 4A). The two dashed lines indicate two special λ values: λ_min_ on the left and λ_1se_ on the right. The λ values between these two values was considered to be appropriate. The model constructed by λ_1se_ was the simplest, that is, it used a small number of genes, while λ_min_ had a higher accuracy rate and used a larger number of genes. Hence, λ_min_ was selected to build the model for accuracy in our study.

The final model contained seven PALs, namely, *CTD-2540B15.13, LINC00893, MIR503HG, RP11-356I2.4, RP11-386G11.10, TCL6*, and *VLDLR-AS1*. A total of 418 TCGA samples were divided into two risk groups, of which 201 were high risk (≥the optimal cut point) and 217 were low risk (<the optimal cutoff point). The results revealed that OS in the high-risk group was markedly lower than that in the low-risk group (Figure 4B). The heat map and RS distribution map of seven lncRNA expression values in each sample were drawn as shown in Figure 4C, which showed that the lower the expression level of the seven PALs, the higher the RS and the shorter survival time. Two GEO datasets were utilized to verify the risk model according to the same method described above, and the K–M curve proved the validity of the model constructed by the seven PALs in survival prediction (Figure 4D).

Univariate and multivariate Cox regression analyses of risk model, grade, Figo stage, and age for TCGA and GEO datasets demonstrated that the risk model was an independent risk factor for OC patients (Table 3). The 1-, 3-, and 5-year survival ROC curves predicted by the risk model were drawn (Figures 4E,F). To better predict prognosis at 1-, 3-, and 5-year OS of OC patients, we constructed a nomogram of variables such as the risk score, grade, and Figo stage (Figures 4G,H).

**Table 3:** Univariate and multivariate Cox analyses of risk signature in TCGA and GEO dataset.

**Variables**	**Univariate**			**Multivariate**		
	**Coefficient**	**HR (95% CI)**	***P-value***	**Coefficient**	**HR (95% CI)**	***P-value***
**TCGA dataset**
Risk score	1.038	2.824 (1.98–4.026)	<0.001	0.887	2.427 (1.601–3.681)	<0.001
Age	0.023	1.023 (1.01–1.036)	0.001	0.024	1.024 (1.011–1.038)	<0.001
Grade	0.119	1.126 (0.786–1.614)	0.517	−0.011	0.989 (0.684–1.429)	0.952
Figo stage	0.178	1.195 (1.017–1.405)	0.031	0.169	1.184 (0.996–1.407)	0.056
**GEO dataset**
Risk score	1.000	2.718 (1.173–6.3)	0.020	0.816	2.262 (0.96–5.328)	0.032
Grade	0.481	1.618 (1.122–2.335)	0.010	0.373	1.452 (0.987–2.136)	0.058
Figo stage	0.313	1.368 (0.953–1.963)	0.090	0.236	1.267 (0.859–1.868)	0.233

**Figure 5:**
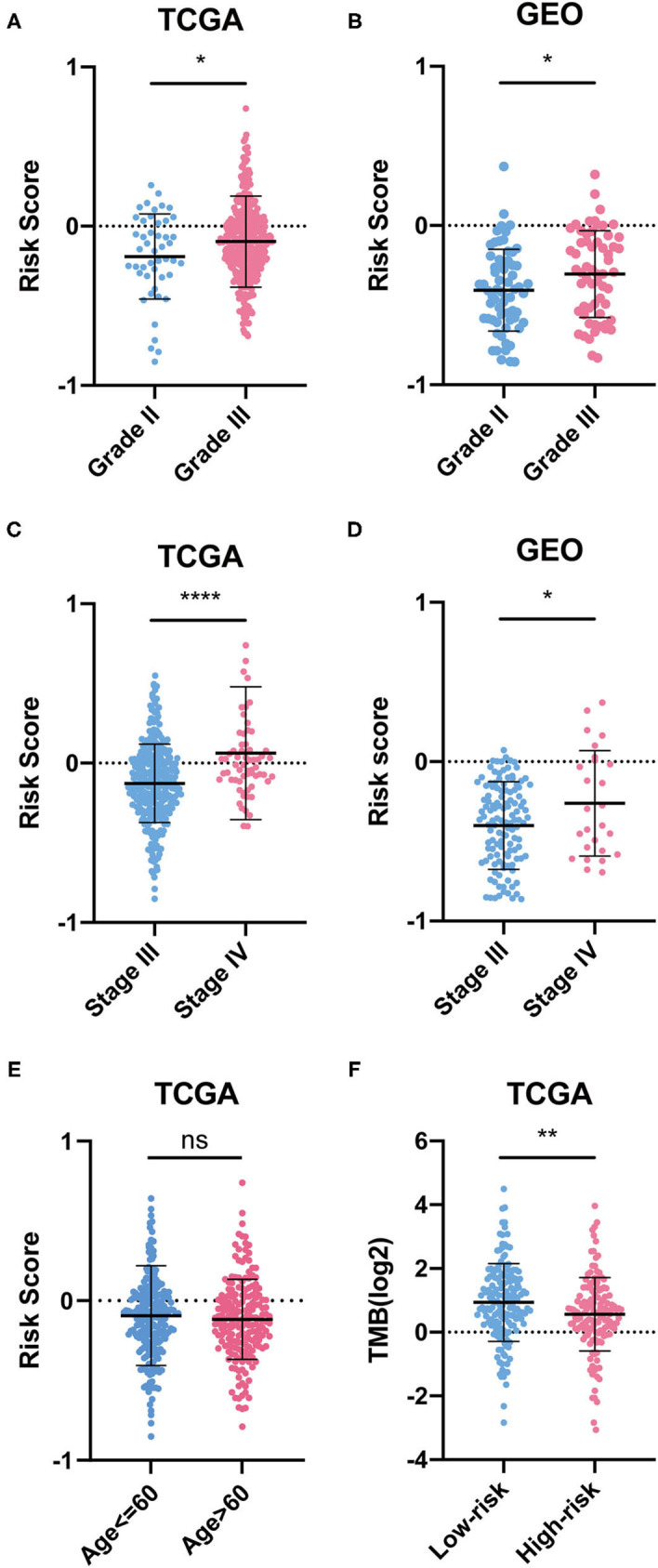
Difference between groups. **(A–E)** Risk scores of clinical indicators. **(F)** TMB between risk groups.

We also found that OC patients with grade III and stage IV OC had higher RSs, while RS was not related to age (Figures 5A–E). As for TMB, OC patients in the low-risk group had lower TMB scores, which indicated that they may be more likely to respond to immunotherapy (Figure 5F).

### Construction of a PAL-Associated ceRNA Network

**Figure 6:**
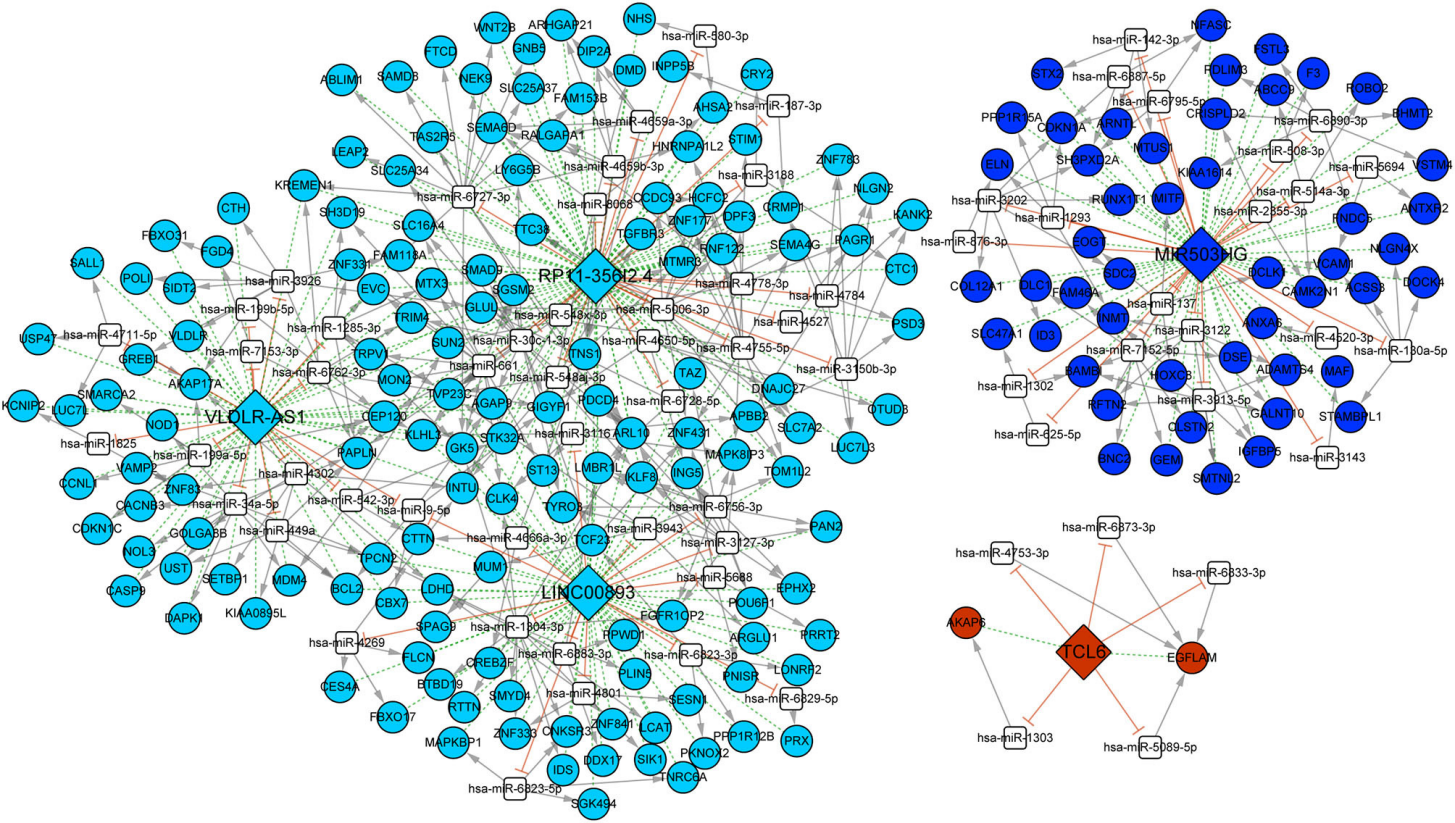
ceRNA network of five PALs. Different colors represented different modules. Diamonds represented lncRNAs, circles represented mRNAs, and white squares represented predicted miRNAs. Dotted green lines represented coexpression of lncRNAs and mRNAs, gray arrows represented miRNA regulated mRNAs, and orange T-shaped lines represented competing binding mRNAs.

We predicted 19,630 miRNA–mRNA pairs and 129 PAL–miRNA relationship pairs. PALs and mRNAs that were regulated by the same miRNA were screened, and the positively coexpressed PAL–mRNA pairs were combined. Finally, 347 PAL–miRNA–mRNA relationship pairs were obtained, including 5 PALs (*TCL6, VLDLR-AS1, RP11-356I2.4, LINC00893*, and *MIR503HG*), 70 miRNAs, and 199 mRNAs (Figure 6). There were 71 PAL–miRNA relationship pairs, 341 miRNA–mRNA relationship pairs, and 242 PAL–mRNA coexpression relationships in the ceRNA network, which could be used to explore the molecular mechanisms involved in the development of OC.

### *In vitro* Assays

**Figure 7:**
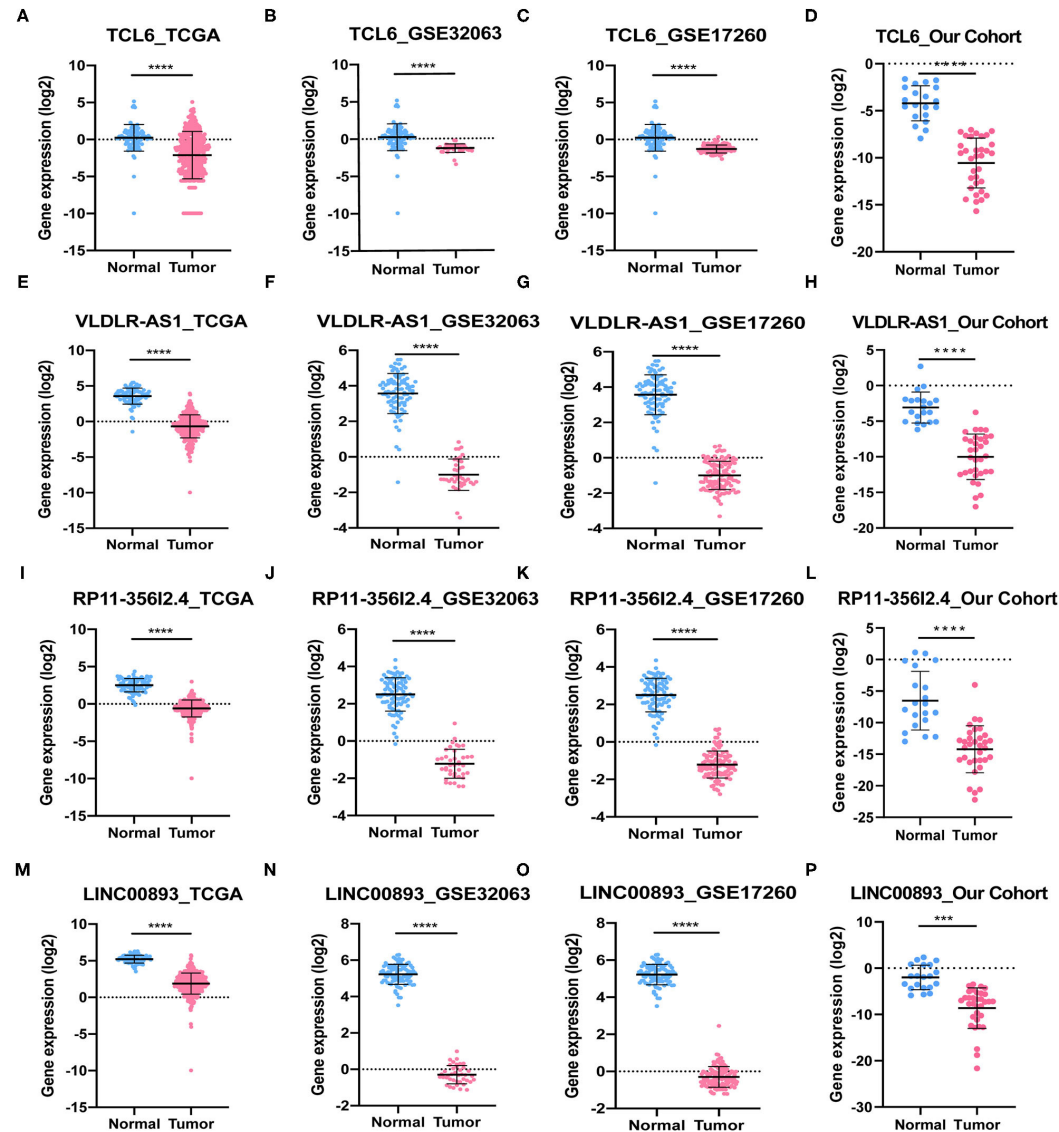
The expression of four PALs in normal and OC samples. **(A–D)** TCL6. **(E–H)** VLDLR-AS1. **(I–L)** RP11-356I2.4. **(M–P)** LINC00893. ****P < 0.0001. ***P < 0.001.

**Figure 8:**
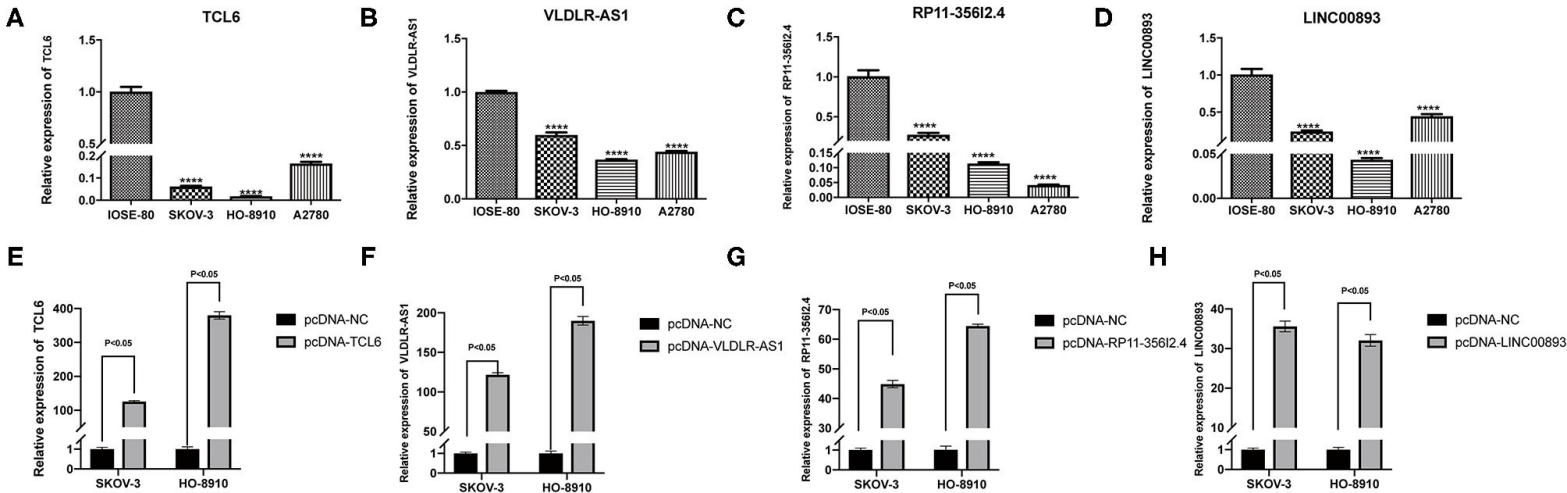
**(A–D)** The expression of four candidate PALs in IOSE-80 and OC cells. **(E–H)** The expression level of four candidate PALs under SKOV-3 and HO-8910 transfection.

Among the five PALs in the ceRNA network we constructed, the functions of lncRNA MIR503HG involved in OC have been investigated (Zhu et al., 2020); hence, the remaining four PALs were preliminarily selected as candidate molecules to perform cell function assays *in vitro*. Analysis of TCGA dataset (418 OC samples), GSE32063 (40 OC samples), GSE17260 (110 OC samples), and our cohort (33 OC samples) showed that the four PALs were evidently downregulated in OC tissues when compared with normal controls (Figures 7A–P). The expression of the four PALs in three OC cell lines (SKOV-3, HO8910, and A2780) and the normal ovarian epithelial cell line IOSE-80 was detected. As shown in Figures 8A–D, the expression of the four PALs was significantly lower in OC cells than in IOSE-80 cells (*p* < 0.05).

**Figure 9:**
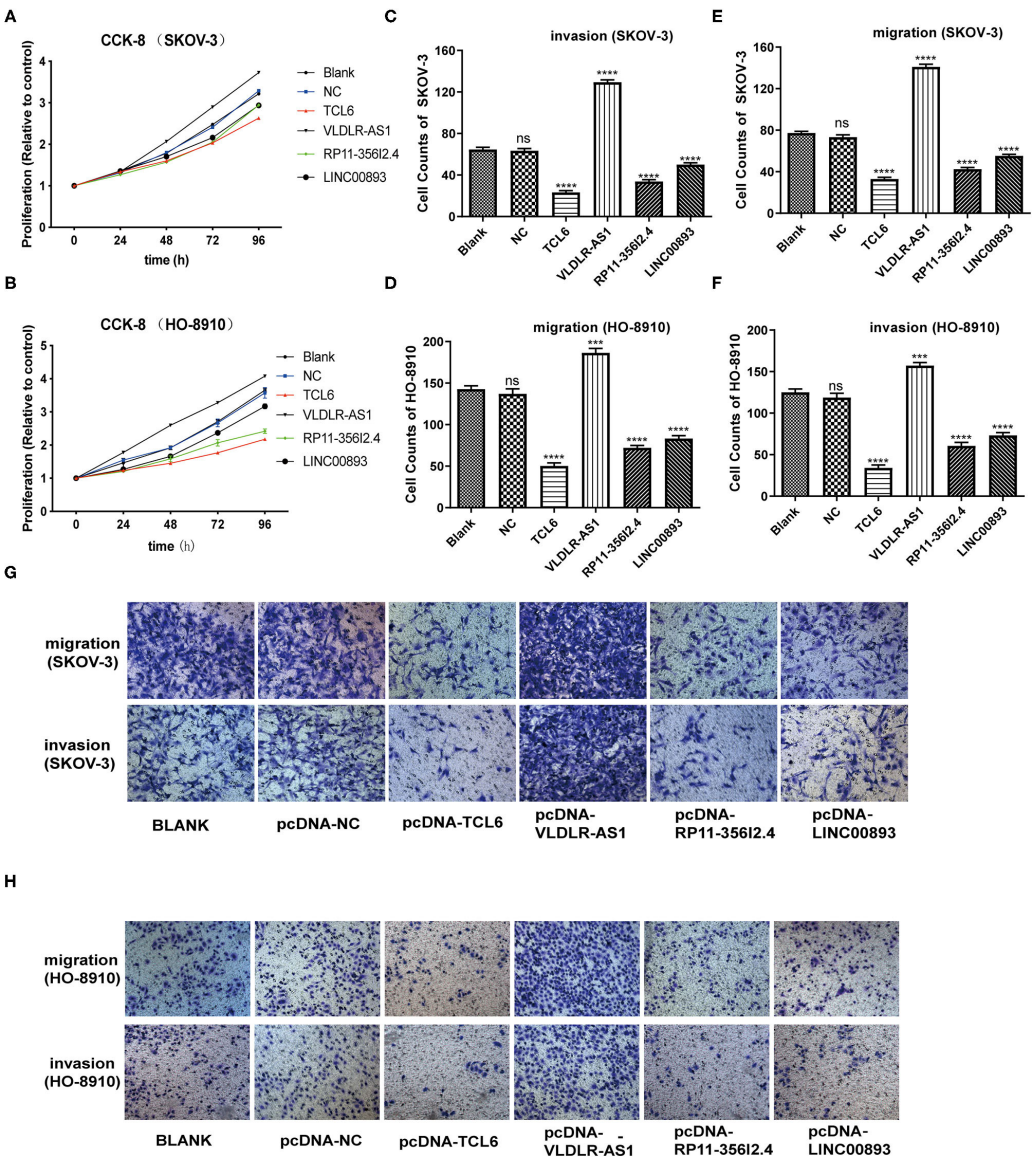
CCK-8 and transwell assays. **(A)** CCK-8 assays of transfected SKOV-3. **(B)** CCK-8 assays of transfected HO-8910 cells. **(C–H)** Transwell assays of transfected SKOV-3 or HO-8910 cells.

The K–M survival curves confirmed that higher expression of the four PALs was associated with better OS, which indicated that they may serve as tumor suppressor genes for OC (Supplementary Figure 2). Subsequently, we confirmed that the expression levels of the four PALs increased following plasmid transfection in SKOV-3 and HO-8910 cells (Figures 8E–H). Later, data from the CCK-8 assay illustrated that overexpression of TCL6, RP11-356I2.4, and LINC00893 reduced the viability of OC cells (Figures 9A,B). Nevertheless, the proliferative abilities of SKOV-3 and HO-8910 cells increased after VLDLR-AS1 overexpression (Figures 9A,B). In addition, the number of migrated and invaded OC cells declined with the overexpression of TCL6, RP11-356I2.4, and LINC00893, according to data from transwell assays (Figures 9C–H). On the contrary, overexpression of VLDLR-AS1 increased the invasive and metastatic abilities of SKOV-3 and HO-8910 cells (Figures 9C–H).

## Discussion

Recent studies have shown that lncRNAs play an important role in regulating the growth, division, metastasis, invasion, proliferation, and drug resistance of OC cells (Braga et al., 2020). Abnormal expression of some lncRNAs in OC may provide important reference information for the diagnosis, treatment, and prognosis of patients (Salamini-Montemurri et al., 2020). However, compared with miRNA, the study of lncRNAs for OC is still in its infancy (Razavi et al., 2021). Therefore, it is necessary to further study lncRNAs in OC. Previous studies have inspired us to explore potential prognosis-associated lncRNAs in OC.

WGCNA is the most representative systems biology algorithm based on transcriptome data to construct gene coexpression networks (Zhang and Horvath, 2005; Langfelder and Horvath, 2008). Using WGCNA, information on gene expression in biological systems can be analyzed quantitatively and at different levels. Although previous studies have applied WGCNA to established an lncRNA-associated signature in malignant tumors (Gong and Ning, 2020; Han P. et al., 2020; Li et al., 2020; Tian et al., 2021; Yuan et al., 2021), research on its application in OC is sparse. In our study, seven stable modules with highly correlated 3,560 genes and correlations with specific clinical factors were clustered. The prognostic risk model based on seven PALs could divide OC patients into two risk groups according to optimal cutoff point, which was validated using two datasets from GEO. After construction of the ceRNA network, four PALs (*TCL6, VLDLR-AS1, RP11-356I2.4*, and *LINC00893*) in the network were selected for further cell function assays. In the WGCNA analysis, *TCL6* was gathered into the brown module, and *VLDLR-AS1, RP11-356I2.4*, and *LINC00893* were gathered into the turquoise module. Therefore, *TCL6* may be related to the clinical stage and histological grade of OC. Meanwhile, *VLDLR-AS1, RP11-356I2.4*, and *LINC00893* may be associated with the clinical stage. The KEGG pathway of coexpression analysis showed that three PALs were enriched in several biological functions of OC, such as glutamatergic synapse, inflammatory mediator regulation of TRP channels, and ovarian steroidogenesis, which indicated their potential therapeutic targets.

A previous study revealed that lncRNA *miR503HG* was downregulated in OC, and downregulation of *miR503HG* predicted poor survival of OC patients (Zhu et al., 2020), which coincided with *miR503HG* in our prognostic risk model signature. Coincidentally, the expression of *MIR503HG* was decreased in colon cancer (Chuo et al., 2019; Han H. et al., 2020), triple-negative breast cancer (Fu et al., 2019; Tuluhong et al., 2020), non-small cell lung cancer (Lin et al., 2019; Dao et al., 2020; Xu et al., 2020), cervical squamous cell carcinoma (Zhao et al., 2020), bladder cancer (Qiu et al., 2019), and hepatocellular carcinoma (Wang et al., 2018). *MIR503HG* was also proved to serve as a tumor suppressor in *in vivo* experiment. *TCL6* had been demonstrated to be a potential tumor suppressor in breast cancer (Zhang et al., 2020), hepatocellular carcinoma (Luo et al., 2020), renal cell carcinoma (Yang et al., 2018; Kulkarni et al., 2021), and B-cell acute lymphoblastic leukemia (Cuadros et al., 2019). Overexpression of *TCL6* in corresponding cancer cell lines impairs their oncogenic functions, such as cell proliferation and migration/invasion. *RP11-356I2.4* (also known as *WAKMAR2, lnc-TNFAIP3*, or *LOC100130476*) has been shown to act as a tumor suppressor gene in esophageal cancer and gastric cardia adenocarcinoma. In addition, upregulation of *RP11-356I2.4* led to the inhibition of proliferation and invasiveness of cancer cells (Guo et al., 2016a,b). *LINC00893* was lowly expressed in thyroid carcinoma tissues and papillary thyroid cancer (PTC) cells. Furthermore, *LINC00893* overexpression abrogated the proliferation and migration abilities of PTC cells (Li et al., 2021). In our prognostic risk model signature, lncRNA *TCL6, RP11-356I2.4*, and *LINC00893* were all downregulated in OC cells and tissues. In addition, downregulation of these genes indicated poor OS in patients with OC. Through gain-of-function assays, we determined that the overexpression of the three PALs restrained the proliferation and migration abilities of OC cells, which would fill the gap in their study in OC. Hence, these three molecules are also tumor suppressor genes for OC, suggesting their potential as biomarkers and therapeutic targets. Previous studies and our cell function assays further illustrate the accuracy of our risk score model. However, more in-depth molecular mechanisms are yet to be studied by subsequent researchers.

Although lncRNA *VLDLR-AS1* was downregulated and downregulation of VLDLR-AS1 indicated poor survival in OC, *VLDLR-AS1* seemed to be an oncogene in terms of OC cellular function. Previous studies showed that *VLDLR-AS1* is highly expressed in esophageal squamous cell carcinoma (Chen et al., 2019) and hepatocellular carcinoma (HCC) (Yang et al., 2017). Knocking down the expression of *VLDLR-AS1* inhibited the proliferation of HCC cells, which was consistent with our cell function assays. The differences in *VLDLR-AS1* expression were not consistent with the cell function experiment in OC, which is worthy of further study by subsequent researchers.

There are some limitations to our study. First, there were only 33 OC patients without OS in our cohort; hence, more time and more samples are needed for follow-up. Second, the cell function assays of four candidate lncRNAs were preliminary, which requires further investigation to provide a better understanding.

## Conclusions

In summary, we performed weighted gene coexpression analysis on differentially expressed genes obtained from datasets to screen for modules related to clinical phenotypes and established a seven-PAL-based signature with a prognostic value for OC, which could stratify OC patients into two risk groups with significant differences in prognosis. Two additional datasets were used to verify the accuracy of the model. Meanwhile, four candidate PALs were selected to perform cell function assays, which need further studies of subsequent researchers.

## Data Availability Statement

The datasets presented in this study can be found in online repositories. The names of the repository/repositories and accession number(s) can be found in the article/Supplementary Material.

## Ethics Statement

The studies involving human participants were reviewed and approved by Affiliated Hangzhou Hospital of Nanjing Medical University. The patients/participants provided their written informed consent to participate in this study.

## Author Contributions

JT and ZZ conceived, designed, and supervised the study. JZ performed data analysis and drafted the manuscript. JG helped revise the manuscript. GX, HZ, and BC collected the data. All the authors reviewed and approved the final manuscript.

## Conflict of Interest

The authors declare that the research was conducted in the absence of any commercial or financial relationships that could be construed as a potential conflict of interest.

## Supplementary Material

Supplementary Figure 1 Correlation analysis results between WGCNA modules and clinical phenotypes. **(A)** The upper number represented the correlation coefficient, and the lower bracket number represented the significance *P*-value. The modules related to clinical phenotype were mined. **(B)** Age at initial pathologic diagnosis. **(C)** Clinical stage. **(D)** Lymphatic invasion. **(E)** Neoplasm histologic grade. **(F)** Tumor residual disease. **(G)** Venous invasion. **(H)** Vital status.

Supplementary Figure 2 K-M survival curves of 24 lncRNAs.

Supplementary Table 1 Baseline characteristic of patients with ovarian cancer.

## Abbreviations

GEO: Gene Expression Omnibus
HR: hazard ratio
K–M: Kaplan–Meier
lncRNA: long non-coding RNA
OC: ovarian cancer
OS: overall survival
PAL: prognosis-associated lncRNA
ROC: receiver operating characteristic
TCGA: The Cancer Genome Atlas
WGCNA: weighted gene coexpression analysis.

## References

[B1] Barrett T. Suzek T. O. Troup D. B. Wilhite S. E. Ngau W. C. Ledoux P.. (2005). NCBI GEO: mining millions of expression profiles–database and tools. Nucleic Acids Res. 33, D562–D566. 10.1093/nar/gki022 15608262 PMC539976

[B2] Braga E. A. Fridman M. V. Moscovtsev A. A. Filippova E. A. Dmitriev A. A. Kushlinskii N. E. (2020). LncRNAs in ovarian cancer progression, metastasis, and main pathways: ceRNA and alternative mechanisms. Int. J. Mol. Sci. 21:8855. 10.3390/ijms21228855 33238475 PMC7700431

[B3] Chen G. Y. Zhang Z. S. Chen Y. Li Y. (2021). Long non-coding RNA SNHG9 inhibits ovarian cancer progression by sponging microRNA-214-5p. Oncol. Lett. 21:80. 10.3892/ol.2020.12341 33363617 PMC7723070

[B4] Chen J. Li X. Yang L. Zhang J. (2020). Long Non-coding RNA LINC01969 promotes ovarian cancer by regulating the miR-144-5p/LARP1 axis as a competing endogenous RNA. Front. Cell Dev. Biol. 8:625730. 10.3389/fcell.2020.595585 33614632 PMC7889973

[B5] Chen Y. Liu L. Li J. Du Y. Wang J. Liu J. (2019). Effects of long noncoding RNA (linc-VLDLR) existing in extracellular vesicles on the occurrence and multidrug resistance of esophageal cancer cells. Pathol. Res. Pract. 215, 470–477. 10.1016/j.prp.2018.12.033 30606658

[B6] Chuo D. Liu F. Chen Y. Yin M. (2019). LncRNA MIR503HG is downregulated in Han Chinese with colorectal cancer and inhibits cell migration and invasion mediated by TGF-β2. Gene 713:143960. 10.1016/j.gene.2019.143960 31278965

[B7] Cintolo-Gonzalez J. A. Braun D. Blackford A. L. Mazzola E. Acar A. Plichta J. K.. (2017). Breast cancer risk models: a comprehensive overview of existing models, validation, and clinical applications. Breast Cancer Res. Treat. 164, 263–284. 10.1007/s10549-017-4247-z 28444533

[B8] Cuadros M. Andrades Á. Coira I. F. Baliñas C. Rodríguez M. I. Álvarez-Pérez J. C.. (2019). Expression of the long non-coding RNA TCL6 is associated with clinical outcome in pediatric B-cell acute lymphoblastic leukemia. Blood Cancer J. 9:93. 10.1038/s41408-019-0258-9 31767830 PMC6877621

[B9] Dao R. Wudu M. Hui L. Jiang J. Xu Y. Ren H.. (2020). Knockdown of lncRNA MIR503HG suppresses proliferation and promotes apoptosis of non-small cell lung cancer cells by regulating miR-489-3p and miR-625-5p. Pathol. Res. Pract. 216:152823. 10.1016/j.prp.2020.152823 31983569

[B10] Eisenhauer E. A. (2017). Real-world evidence in the treatment of ovarian cancer. Ann. Oncol. 28(Suppl. 8), viii61–viii65. 10.1093/annonc/mdx443 29232466

[B11] Engebretsen S. Bohlin J. (2019). Statistical predictions with glmnet. Clin. Epigenetics 11:123. 10.1186/s13148-019-0730-1 31443682 PMC6708235

[B12] Fu J. Dong G. Shi H. Zhang J. Ning Z. Bao X.. (2019). LncRNA MIR503HG inhibits cell migration and invasion via miR-103/OLFM4 axis in triple negative breast cancer. J. Cell. Mol. Med. 23, 4738–4745. 10.1111/jcmm.14344 31062436 PMC6584514

[B13] Goldman M. J. Craft B. Hastie M. Repečka K. McDade F. Kamath A.. (2020). Visualizing and interpreting cancer genomics data via the Xena platform. Nat. Biotechnol. 38, 675–678. 10.1038/s41587-020-0546-8 32444850 PMC7386072

[B14] Gong X. Ning B. (2020). Five lncRNAs associated with prostate cancer prognosis identified by coexpression network analysis. Technol. Cancer Res. Treat. 19:1533033820963578. 10.1177/1533033820963578 33084528 PMC7785998

[B15] Guo W. Dong Z. Shi Y. Liu S. Liang J. Guo Y.. (2016a). Aberrant methylation-mediated downregulation of long noncoding RNA LOC100130476 correlates with malignant progression of esophageal squamous cell carcinoma. Dig. Liver Dis. 48, 961–969. 10.1016/j.dld.2016.05.010 27338851

[B16] Guo W. Dong Z. Shi Y. Liu S. Liang J. Guo Y.. (2016b). Methylation-mediated downregulation of long noncoding RNA LOC100130476 in gastric cardia adenocarcinoma. Clin. Exp. Metastasis 33, 497–508. 10.1007/s10585-016-9794-x 27189370

[B17] Han H. Li H. Zhou J. (2020). Long non-coding RNA MIR503HG inhibits the proliferation, migration, and invasion of colon cancer cells via miR-107/Par4 axis. Exp. Cell Res. 395:112205. 10.1016/j.yexcr.2020.112205 32738347

[B18] Han P. Yang H. Li X. Wu J. Wang P. Liu D.. (2020). Identification of a novel cancer stemness-associated ceRNA axis in lung adenocarcinoma via stemness indices analysis. Oncol. Res. 10.3727/096504020X16037124605559. [Epub ahead of print]. 33106209 PMC8420898

[B19] Harrow J. Frankish A. Gonzalez J. M. Tapanari E. Diekhans M. Kokocinski F.. (2012). GENCODE: the reference human genome annotation for The ENCODE Project. Genome Res. 22, 1760–1774. 10.1101/gr.135350.111 22955987 PMC3431492

[B20] Hsu S. D. Lin F. M. Wu W. Y. Liang C. Huang W. C. Chan W. L.. (2011). miRTarBase: a database curates experimentally validated microRNA-target interactions. Nucleic Acids Res. 39, D163–D169. 10.1093/nar/gkq1107 21071411 PMC3013699

[B21] Kaldawy A. Segev Y. Lavie O. Auslender R. Sopik V. Narod S. A. (2016). Low-grade serous ovarian cancer: a review. Gynecol. Oncol. 143, 433–438. 10.1016/j.ygyno.2016.08.320 27581327

[B22] Kohl M. Wiese S. Warscheid B. (2011). Cytoscape: software for visualization and analysis of biological networks. Methods Mol. Biol. 696, 291–303. 10.1007/978-1-60761-987-1_18 21063955

[B23] Kopp F. Mendell J. T. (2018). Functional classification and experimental dissection of long noncoding RNAs. Cell 172, 393–407. 10.1016/j.cell.2018.01.011 29373828 PMC5978744

[B24] Kulkarni P. Dasgupta P. Hashimoto Y. Shiina M. Shahryari V. Tabatabai Z. L.. (2021). A lncRNA TCL6-miR-155 interaction regulates the Src-Akt-EMT network to mediate kidney cancer progression and metastasis. Cancer Res. 81, 1500–1512. 10.1158/0008-5472.CAN-20-0832 33500248 PMC7969457

[B25] Langfelder P. Horvath S. (2008). WGCNA: an R package for weighted correlation network analysis. BMC Bioinformatics 9:559. 10.1186/1471-2105-9-559 19114008 PMC2631488

[B26] Li J. Zhou J. Kai S. Wang C. Wang D. Jiang J. (2020). Network-based coexpression analysis identifies functional and prognostic long noncoding RNAs in hepatocellular carcinoma. Biomed Res. Int. 2020:1371632. 10.1155/2020/1371632 33083449 PMC7559504

[B27] Li N. Zhan X. (2019). Identification of clinical trait-related lncRNA and mRNA biomarkers with weighted gene co-expression network analysis as useful tool for personalized medicine in ovarian cancer. EPMA J. 10, 273–290. 10.1007/s13167-019-00175-0 31462944 PMC6695468

[B28] Li S. Zhang Y. Dong J. Li R. Yu B. Zhao W.. (2021). LINC00893 inhibits papillary thyroid cancer by suppressing AKT pathway via stabilizing PTEN. Cancer Biomark. 30, 277–286. 10.3233/CBM-190543 32924982 PMC12499967

[B29] Lin H. Li P. Zhang N. Cao L. Gao Y. F. Ping F. (2019). Long non-coding RNA MIR503HG serves as a tumor suppressor in non-small cell lung cancer mediated by wnt1. Eur. Rev. Med. Pharmacol. Sci. 23, 10818–10826. 10.26355/eurrev_201912_19785 31858550

[B30] Luo L. H. Jin M. Wang L. Q. Xu G. J. Lin Z. Y. Yu D. D.. (2020). Long noncoding RNA TCL6 binds to miR-106a-5p to regulate hepatocellular carcinoma cells through PI3K/AKT signaling pathway. J. Cell. Physiol. 235, 6154–6166. 10.1002/jcp.29544 32020591

[B31] Paraskevopoulou M. D. Vlachos I. S. Karagkouni D. Georgakilas G. Kanellos I. Vergoulis T.. (2016). DIANA-LncBase v2: indexing microRNA targets on non-coding transcripts. Nucleic Acids Res. 44, D231–D238. 10.1093/nar/gkv1270 26612864 PMC4702897

[B32] Qiu F. Zhang M. R. Zhou Z. Pu J. X. Zhao X. J. (2019). lncRNA MIR503HG functioned as a tumor suppressor and inhibited cell proliferation, metastasis and epithelial-mesenchymal transition in bladder cancer. J. Cell. Biochem. 120, 10821–10829. 10.1002/jcb.28373 30672010

[B33] Razavi Z. S. Tajiknia V. Majidi S. Ghandali M. Mirzaei H. R. Rahimian N.. (2021). Gynecologic cancers and non-coding RNAs: epigenetic regulators with emerging roles. Crit. Rev. Oncol. Hematol. 157:103192. 10.1016/j.critrevonc.2020.103192 33290823

[B34] Ritchie M. E. Phipson B. Wu D. Hu Y. Law C. W. Shi W.. (2015). limma powers differential expression analyses for RNA-sequencing and microarray studies. Nucleic Acids Res. 43:e47. 10.1093/nar/gkv007 25605792 PMC4402510

[B35] Salamini-Montemurri M. Lamas-Maceiras M. Barreiro-Alonso A. Vizoso-Vázquez Á. Rodríguez-Belmonte E. Quindós-Varela M.. (2020). The challenges and opportunities of LncRNAs in ovarian cancer research and clinical use. Cancers (Basel) 12:1020. 10.3390/cancers12041020 32326249 PMC7225988

[B36] Stewart C. Ralyea C. Lockwood S. (2019). Ovarian cancer: an integrated review. Semin. Oncol. Nurs. 35, 151–156. 10.1016/j.soncn.2019.02.001 30867104

[B37] Sun Q. Zhao H. Zhang C. Hu T. Wu J. Lin X.. (2017). Gene co-expression network reveals shared modules predictive of stage and grade in serous ovarian cancers. Oncotarget 8, 42983–42996. 10.18632/oncotarget.17785 28562334 PMC5522121

[B38] Tammemägi M. C. Ten Haaf K. Toumazis I. Kong C. Y. Han S. S. Jeon J.. (2019). Development and validation of a multivariable lung cancer risk prediction model that includes low-dose computed tomography screening results: a secondary analysis of data From the national lung screening trial. JAMA Netw. Open 2:e190204. 10.1001/jamanetworkopen.2019.0204 30821827 PMC6484623

[B39] Tian F. Zhao J. Fan X. Kang Z. (2017). Weighted gene co-expression network analysis in identification of metastasis-related genes of lung squamous cell carcinoma based on the Cancer Genome Atlas database. J. Thorac. Dis. 9, 42–53. 10.21037/jtd.2017.01.04 28203405 PMC5303106

[B40] Tian S. Zhang M. Ma Z. (2021). An edge-based statistical analysis of long non-coding RNA expression profiles reveals a negative association between Parkinson's disease and colon cancer. BMC Med. Genomics 14:36. 10.1186/s12920-021-00882-6 33531021 PMC7851899

[B41] Tuluhong D. Dunzhu W. Wang J. Chen T. Li H. Li Q.. (2020). Prognostic value of differentially expressed lncrnas in triple-negative breast cancer: a systematic review and meta-analysis. Crit. Rev. Eukaryot. Gene Expr. 30, 447–456. 10.1615/CritRevEukaryotGeneExpr.2020035836 33389881

[B42] Van Cott C. (2020). Cancer genetics. Surg. Clin. North Am. 100, 483–498. 10.1016/j.suc.2020.02.012 32402295

[B43] Wang H. Liang L. Dong Q. Huan L. He J. Li B.. (2018). Long noncoding RNA miR503HG, a prognostic indicator, inhibits tumor metastasis by regulating the HNRNPA2B1/NF-κB pathway in hepatocellular carcinoma. Theranostics 8, 2814–2829. 10.7150/thno.23012 29774077 PMC5957011

[B44] Wang J. Y. Lu A. Q. Chen L. J. (2019). LncRNAs in ovarian cancer. Clin. Chim. Acta 490, 17–27. 10.1016/j.cca.2018.12.013 30553863

[B45] Xu S. Zhai S. Du T. Li Z. (2020). LncRNA MIR503HG inhibits non-small cell lung cancer cell proliferation by inducing cell cycle arrest through the downregulation of cyclin D1. Cancer Manag. Res. 12, 1641–1647. 10.2147/CMAR.S227348 32184667 PMC7062398

[B46] Yang K. Lu X. F. Luo P. C. Zhang J. (2018). Identification of six potentially long noncoding RNAs as biomarkers involved competitive endogenous RNA in clear cell renal cell carcinoma. Biomed Res. Int. 2018:9303486. 10.1155/2018/9303486 30406146 PMC6201332

[B47] Yang N. Li S. Li G. Zhang S. Tang X. Ni S.. (2017). The role of extracellular vesicles in mediating progression, metastasis and potential treatment of hepatocellular carcinoma. Oncotarget 8, 3683–3695. 10.18632/oncotarget.12465 27713136 PMC5356911

[B48] Yoshihara K. Tajima A. Yahata T. Kodama S. Fujiwara H. Suzuki M.. (2010). Gene expression profile for predicting survival in advanced-stage serous ovarian cancer across two independent datasets. PLoS ONE 5:e9615. 10.1371/journal.pone.0009615 20300634 PMC2837379

[B49] Yoshihara K. Tsunoda T. Shigemizu D. Fujiwara H. Hatae M. Fujiwara H.. (2012). High-risk ovarian cancer based on 126-gene expression signature is uniquely characterized by downregulation of antigen presentation pathway. Clin. Cancer Res. 18, 1374–1385. 10.1158/1078-0432.CCR-11-2725 22241791

[B50] Yu G. Wang L. G. Han Y. He Q. Y. (2012). clusterProfiler: an R package for comparing biological themes among gene clusters. Omics 16, 284–287. 10.1089/omi.2011.0118 22455463 PMC3339379

[B51] Yuan C. Yuan H. Chen L. Sheng M. Tang W. (2021). A novel three-long noncoding RNA risk score system for the prognostic prediction of triple-negative breast cancer. Biomark. Med. 15, 43–55. 10.2217/bmm-2020-0505 33427499

[B52] Zhang B. Horvath S. (2005). A general framework for weighted gene co-expression network analysis. Stat. Appl. Genet. Mol. Biol. 4:17. 10.2202/1544-6115.1128 16646834

[B53] Zhang Y. Li Z. Chen M. Chen H. Zhong Q. Liang L.. (2020). lncRNA TCL6 correlates with immune cell infiltration and indicates worse survival in breast cancer. Breast Cancer 27, 573–585. 10.1007/s12282-020-01048-5 31960363

[B54] Zhao Q. Fan C. (2019). A novel risk score system for assessment of ovarian cancer based on co-expression network analysis and expression level of five lncRNAs. BMC Med. Genet. 20:103. 10.1186/s12881-019-0832-9 31182053 PMC6558878

[B55] Zhao S. Yu M. Wang L. (2020). LncRNA miR503HG regulates the drug resistance of recurrent cervical squamous cell carcinoma cells by regulating miR-155/Caspase-3. Cancer Manag. Res. 12, 1579–1585. 10.2147/CMAR.S225489 32184661 PMC7060770

[B56] Zhu D. Huang X. Liang F. Zhao L. (2020). LncRNA miR503HG interacts with miR-31-5p through multiple ways to regulate cancer cell invasion and migration in ovarian cancer. J. Ovarian Res. 13:3. 10.1186/s13048-019-0599-9 31907059 PMC6945408

