# Supplementary figures and images for: Four Prognosis-Associated lncRNAs Serve as Biomarkers in Ovarian Cancer
Source: Front Genet. 2021 Jul 2;12:672674. doi: 10.3389/fgene.2021.672674 (PMC8336869; doi:10.3389/fgene.2021.672674)

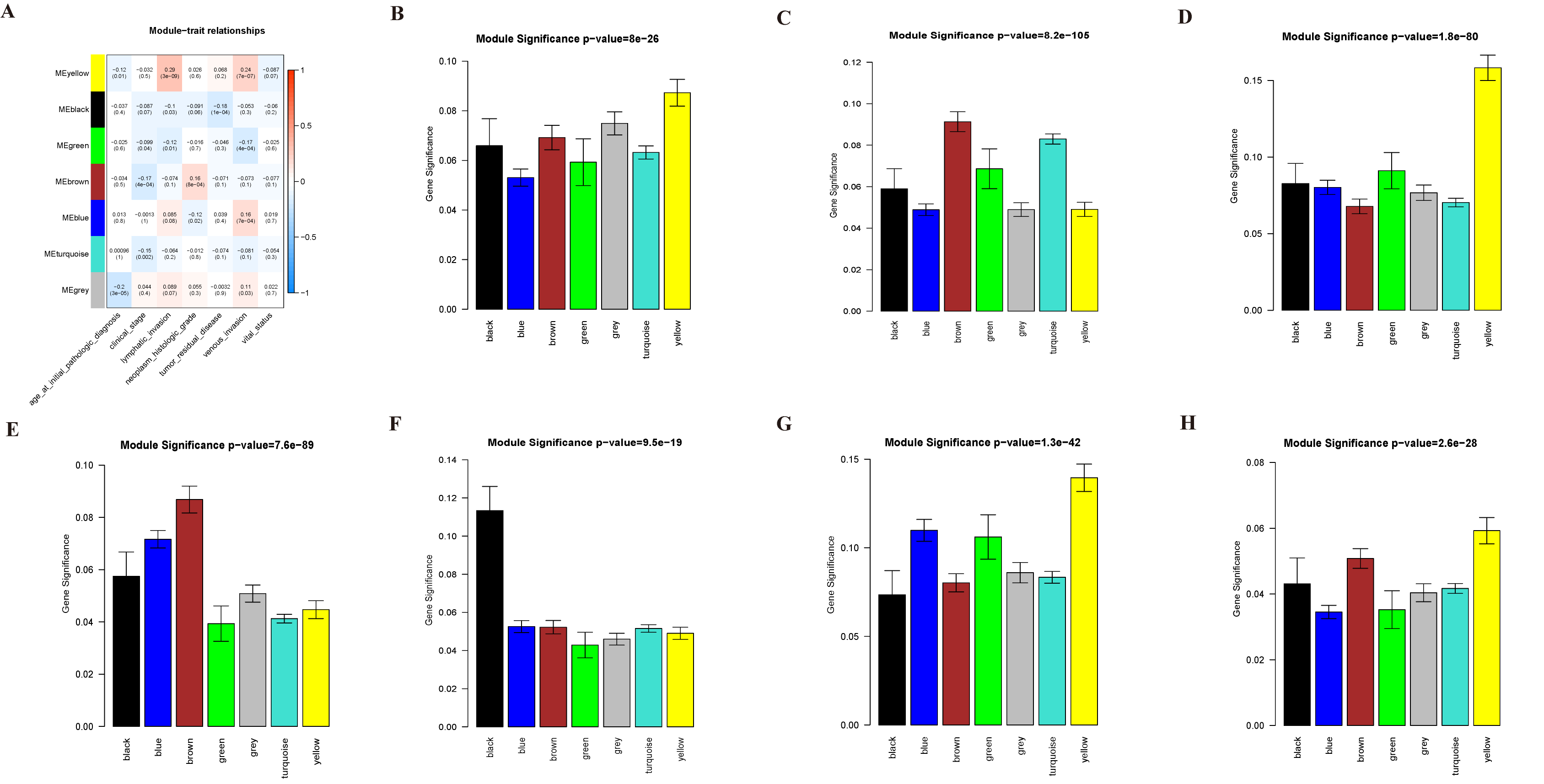

Supplement: Supplementary Figure 1 — Correlation analysis results between WGCNA modules and clinical phenotypes. (A) The upper number represented the correlation coefficient, and the lower bracket number represented the significance P-value. The modules related to clinical phenotype were mined. (B) Age at initial pathologic diagnosis. (C) Clinical stage. (D) Lymphatic invasion. (E) Neoplasm histologic grade. (F) Tumor residual disease. (G) Venous invasion. (H) Vital status. [file Image_1.TIF]

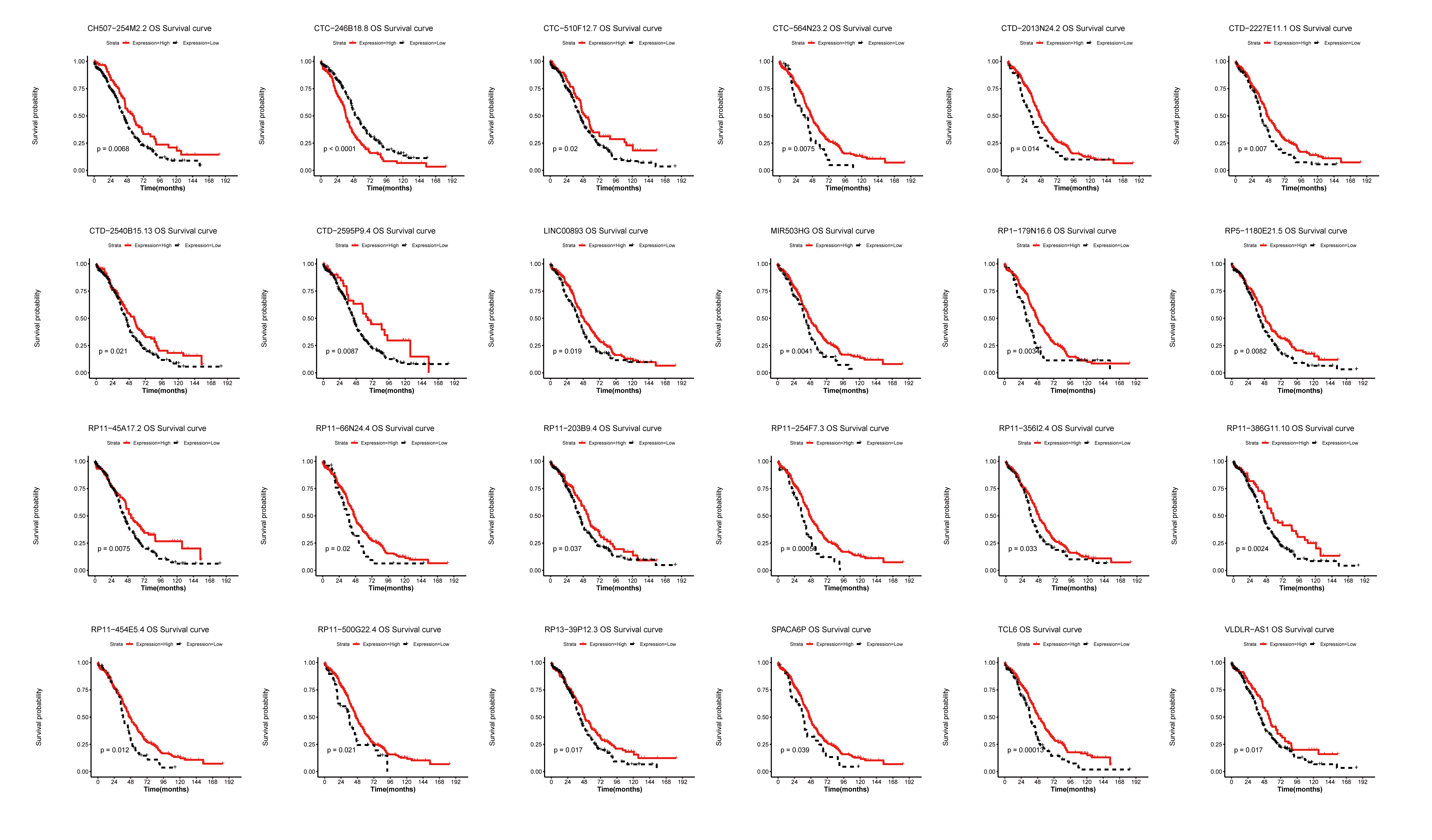

Supplement: Supplementary Figure 2 — K-M survival curves of 24 lncRNAs. [file Image_2.TIF]
